# PTSD assessment in Ukrainian refugees using the PCL-5: linguistic and cultural challenges

**DOI:** 10.3389/fpsyg.2025.1659301

**Published:** 2025-09-16

**Authors:** Sandra Figueiredo, Dimitri Hefter

**Affiliations:** ^1^Department of Psychology, Universidade Autónoma de Lisboa, Lisbon, Portugal; ^2^Department of Psychiatry and Psychotherapy, University of Münster, Münster, Germany; ^3^Central Institute of Mental Health, Mannheim, Germany

**Keywords:** PCL-5, PTSD, Ukrainian refugees, biased back-translation, refugees, psychometrics, bilingualism, semantic similarity metrics

## 1 Introduction

In the aftermath of the full-scale invasion of Ukraine by Russia, multiple language-adapted versions of psychometric instruments have emerged to assess the mental health of near 6 million Ukrainians who sought refuge in European host countries, revealing concerning indicators of poor mental health and challenges related to inclusion behaviors [Sajjad, 2022; UN Refugee Agency (UNHCR), 2022]. Since 2022, there have been several surveys of Ukrainian refugees. However, these studies often lack control over the psychometric properties of the adapted versions used to assess trauma and related symptomatology (Xu et al., 2023).

Recent studies focused on the mental health and wellbeing of refugees from Ukraine, specifically civilians, but the information gathered on trauma and post-traumatic stress disorder (PTSD) remains sparse (Buchcik et al., 2023; Costanza et al., 2022). Mental health indicators suggest a fragile wellbeing environment for these refugees in host countries, which is likely to worsen over time without proper PTSD evaluation (Buchcik et al., 2023; Costanza et al., 2022; Figueiredo et al., 2024; Figueiredo and Petravičiute, 2025; Lotzin et al., 2023). Native language-adapted and cultural-sensitive psychological assessment is crucial for efficient diagnosis and treatment of PTSD symptoms. A well-established instrument for PTSD assessment the PTSD Checklist for DSM-5 (PCL-5; Weathers et al., 2013a), a 20-item self-report of PTSD symptoms according to DMS-5 criteria (American Psychiatry Association, 2013). Several translations in Ukrainian and Russian have been employed amongst Ukrainian refugees. However, the accuracy and validity of these translations remains unclear.

In this study, we address significant gaps in documentation and validation of translated versions of PCL-5. We examined the translation accuracy of its three adaptations in two languages: a Ukrainian (further referred to as UV1; Bezsheiko, 2016) and a Russian (RV; Los Angeles County, Department of Mental Health, 2019) version conducted clinically among Ukrainian refugees in Germany; and another Ukrainian adaptation (UV2; Figueiredo et al., 2024), used in a research setting with Ukrainian refugees in Portugal. We employed both a qualitative and a quantitative approach using bilingual expert ratings as well as novel in this field automated linguistic similarity metrics adapted from machine translations. From these analyses, we derived recommendations for clinical practitioners and researchers.

## 2 The PTSD checklist for DSM-5 (PCL-5)

### 2.1 Psychometric properties of the PCL-5

The PCL-5 has been widely used since its original English version was released in 2013 by Weathers et al. (2013a), with several adaptations following in other languages for both research and clinical purposes. It is based on the diagnostic principles of PTSD in the DSM-5. It consists of 20 items divided into four clusters (B–E) according to the DSM-5 criteria of PTSD: B (items 1–5)—persistent re-experience of the traumatic event; C (items 6–7)—avoidance of trauma-related stimuli; D (items 8–14)—negative changes in cognition and feelings after the trauma; and E (items 15–20)—trauma-related arousal and hyperreactivity. Each item is scored from 0 to 4 based on the participant's response, with the options ranging from “not at all” to “a little bit,” “moderately,” “quite a bit,” and “extremely.” The total possible score ranges from 0 to 80 (Weathers et al., 2013a).

PCL-5 is self-administered and can be finished in well under 20 min, thus being quite resource saving, which is particularly important in acute crises when time and personnel are limited. It has proven to be a reliable instrument with a high internal consistency (α = 0.94; Blevins et al., 2015). Recommended cut-offs range from 30 to 34 (Weathers et al., 2013a; Blevins et al., 2015). However, cut-offs as well as sensitivity and specificity vary strongly depending on the investigated population. Geier et al. (2018) report an astonishingly high sensitivity of 91% and a specificity of 97% for a cut-off of 34 in a US sample of patients following non-intentional traumatic injury. In an Arabic/Kurdish adaptation of PCL-5 employed amongst displaced persons in Kurdistan, a very low cut-off of 23 yielded a sensitivity of 82% and a specificity of 70% (Ibrahim et al., 2018). Boyd et al. (2021) discuss in their study on Canadian psychiatric outpatients two different cut-offs: 33, resulting in a highly sensitive (94%) but unspecific (49%) test; and a cut-off of 45 for optimal ROC with a sensitivity of 82% and specificity of 69%. Thus, PCL-5 compares very well in terms of validity and psychometric properties to other self-administered instruments such as the Primary Care PTSD Screen for DSM-5 (PC-PTSD-5; sensitivity 0.9, specificity 0.8 vs. CAPS-5 in a veteran-sample; Bovin et al., 2021) and clinician-administered instruments such as the PTSD Symptom Scale Interview for DSM-5 (PSSI-5; sensitivity 0.82, specificity 0.71 vs. CAPS-5; Foa et al., 2016). More diagnostic instruments for PSTD diagnosis exist outside of the DSM-5 system. The International Trauma Questionnaire (ITQ, Cloitre et al., 2018) is a self-administered questionnaire for PTSD and complex PTSD according to ICD-11 criteria. Its internal consistently is with α = 0.88 slightly lower than that of PCL-5, but still good. A validated Russian version exists (Padun et al., 2022), but to our knowledge no validated Ukrainian versions, although a DeepL-translated version curated by a bilingual academic is available (OxCADAT Resources). An elder, cross-culturally validated instrument with very high internal validity is the Harvard Trauma Questionnaire (HTQ; Mollica et al., 1992). However, it is outdated being based on DSM-IV and—to the best of our knowledge—lacks a validated Ukrainian adaptation.

Application of PCL-5 should be preceded by the assessment of the trauma A-criterion of PTSD according to DSM-5. The Life Events Checklist for DSM-5 (LEC-5; Weathers et al., 2013b), a 17-item self-report scale to evaluate individuals' prior life events, fulfills this purpose very well. However, its application remains relatively uncommon, which accounts for the limited availability of validated versions of the LEC-5 in languages other than English and a few others.

### 2.2 Limitations of PCL-5

Despite its proven usefulness, several caveats of PCL-5 need to be acknowledged. As stated above, cut-offs and psychometric properties of the PCL-5 are highly heterogeneous depending on the underlying population. Thus, results must be interpreted with cautious consideration of the sociodemographics and disease burden of the investigated sample. Secondly, PTBS A-criterion must be fulfilled based on a separate diagnostic instrument or clinical exploration. Disregarding this step will lead to increased false-positive rates through the risk of assessing the general severity of the psychological burden, possibly unrelated to a PTSD diagnosis. Thirdly, most of PCL-5 items assess symptoms such as disturbances in sleep, intrusive thoughts, negative changes in cognition and emotional state, vegetative symptoms and hyperarousal, which can also occur in depression and anxiety disorders. Our preliminary unpublished work indicates a very strong correlation between PCL-5 scores and scores in Beck's Depression Inventory (BDI-II) and Beck's Anxiety Inventory (BAI) emphasizing the strong symptomatic overlap. This inevitably leads to reduced specificity of PCL-5 in clinical populations with a high burden of psychiatric disease, as reported by Boyd et al. (2021), which may result in high false positive PTSD diagnoses.

Fourthly, it is not stated in the PCL-5 that the questions refer to a specific traumatic event. When answering the questions, the subject might refer to one or several unrelated life events which often do not fulfill the DSM-5 trauma criterion (all items 1–8) or to their general state which is not related to a specific stressful event (all items 9–20). Thus, one cannot avoid a thorough clinical exploration after the testing in which the clinician must find out which life events the subject refers to and what their answers actually mean. Fifthly, as in every self-administered Likert-type-scale, the individual scores are highly subjective and are strongly affected by the subject's tendencies to aggravation, dissimulation, cognitive distortions, motivation and (mis-)understanding of the items, possible leading to highly variable scores in individuals with an objectively similar syndrome.

Taken together, the PCL-5 is a very useful screening tool for PTSD and is advisable to be included in the diagnostic process, but the PTSD diagnosis should not solely rely on its result. A thorough assessment of the psychopathological findings, clinical exploration of the symptoms, the context, the individual medical (and trauma event) history, the linguistic and social background and needs to be performed; differential diagnoses must be considered.

## 3 PCL-5 adaptations for Ukrainian refugees

### 3.1 General challenges of translation of psychometric instruments

The adaptation of psychological assessment instruments, particularly in cross-cultural studies, typically involves a systematic forward-translation and back-translation process as a standard procedure for clinical and research tools (Forkus et al., 2023; Gadeberg et al., 2017; Thompson et al., 2024). Following the translation phase, the validation process is conducted in three stages: a pilot study to evaluate the instrument's linguistic properties, a main study involving administration to the target population, and test-retest reliability assessments. However, the reliability of such instruments can be compromised if the translation process is not rigorously overseen. Specifically, the lack of oversight by expert committees specialized may lead to deviations in translation quality, thereby affecting the validity and reliability of the instrument (Klotz et al., 2023; Leitgöb et al., 2023). A major issue of translations lies the lack of cross-cultural awareness and knowledge of the psychological assessment process.

### 3.2 Lack of documentation and validation of PCL-5 translations

After its development, the PCL-5 has been validated in several languages including Portuguese, French, Spanish and Arabic as well as in different populations including healthy controls, war veterans, PTSD patients and refugees (Carvalho et al., 2020; Haghish et al., 2024; Ibrahim et al., 2018; Rivest-Beauregard et al., 2022). To our knowledge, there are no other versions with psychometric validation.

We identified several Ukrainian versions of the PCL-5 used internationally. In the previous study of the first author on Ukrainian refugees in Portugal (Figueiredo et al., 2024), the Ukrainian PCL-5 version was adopted from a version provided by the Oxford Health NHS Foundation Trust, based on (Weathers et al., 2013a). While this version has limited references available, it was deemed more reliable in terms of translation and validity, as partially corroborated by Bezsheiko (2016). However, Bezsheiko also notes that the website providing access to the Ukrainian version of the PCL-5 is no longer available. Adding to this inconsistency, studies employing Ukrainian adaptations of the English original (Weathers et al., 2013a) rarely acknowledge source-related limitations, further complicating the assessment of their reliability.

This highlights a significant gap in the documentation regarding the adaptations made by researchers and clinicians to the PCL-5 instrument (Kovács et al., 2023; Lushchak et al., 2024; Zasiekina et al., 2023). Addressing this gap, the present study aims to compare two Ukrainian adaptations and one Russian of the PCL-5 to assess PTSD among Ukrainian refugees in two different hosting countries: the Ukrainian version developed by Bezsheiko (2016), clinically applied to Ukrainian refugees in Germany, and the Ukrainian version by Figueiredo et al. (2024) administered in research context with Ukrainian refugees in Portugal. Additionally, the study will also examine the Russian version of the instrument available online (Los Angeles County, Department of Mental Health, 2019) considering the second author utilized both Ukrainian and Russian versions to accommodate for potential language preferences of Ukrainian refugees in Germany.

### 3.3 Linguistic and cultural diversity of the Ukrainian refugee population

Based on our experience in the validation of psychometric instruments, it is important to emphasize the linguistic variability inherent to the Ukrainian language, which parallels the linguistic variants observed in languages like Portuguese and French (Behr and Shishido, 2016; Borsa et al., 2012. Conversely, when authors of adapted versions assume that a specific language translation and adaptation is suitable for all speakers of that language, this assumption is critically flawed because populations vary based on the regions in which they live, despite sharing a common language (Ashbaugh et al., 2016; Bundgaard and Brøgger, 2019; Hoffman et al., 2022). Professionals working in institutional care settings, particularly those involved in early-stage refugee screening, often lack awareness of this linguistic complexity, which can affect the accuracy and cultural appropriateness of the assessments they conduct (Boettcher et al., 2021; Brücker et al., 2023; Wylie et al., 2018). It is imperative to consider the distinct Ukrainian sociocultural and linguistic context, as variations in language and dialectal differences may substantially impact comprehension and the accuracy of responses to standardized measures (Rizzi et al., 2022; Shibuya et al., 2024).

The phenomenon of code-switching, commonly observed among Ukrainian refugees, warrants particular attention, as it reflects broader linguistic adaptations that may have implications for other bilingual or multilingual populations undergoing similar displacement experiences (Bundgaard and Brøgger, 2019; Urbanaviče et al., 2024; Wylie et al., 2018). Code-switching should be analyzed in the context of insights derived from Ukrainian history, as well as the linguistic and cultural variations between the eastern and western regions of the country. Furthermore, the issue of item differential functioning is often overlooked by researchers and scholars utilizing screening instruments for migrant populations, despite some exceptions (Bockhop et al., 2022; Orlando and Marshall, 2002).

Ukraine is a country with 48 million people and spanning over 1,300 km from east to west and nearly 900 km from north to south with Ukrainian being its official constitutional language. Seventy eight percentage of the population are ethnical Ukrainians, 17% are Russians according to the 2001 Ukraine Census (State Statistics Committee of Ukraine, 2003). Ukraine's language landscape is highly diverse across regions and underwent significant changes over time. In 1989, 64% of the population spoke Ukrainian, 33% Russian as their native language, 3% were bilingual in the 1989 USSR Census (Goskomstat SSSR, 1991). The role of Yiddish severely declined from 14% in the early soviet to 4% in the late soviet era due to decreasing Jewish population (Grenoble, 2003). Following the fall of the USSR and Ukraine's independence, Russian native speakers slightly decreased to 30% (State Statistics Committee of Ukraine, 2003). Full-scale invasion by Russia in 2022 induced a major shift in the language preference of Ukrainians. The preference for Russian as the first language dropped to 13% in a nationally representative survey with ~2,000 respondents in 2024. Interestingly, the Ukrainian speaking population stayed at 63%, while the amount of respondents who considered themselves bilingual increased to 24% (Kulyk, 2024).

Historically, the eastern regions have been under strong influence of the Russian language and culture, the western regions have been influenced by Poland, Moldova and Romania, the northern regions by Poland and Belarus, and the south was affected by Turks and Tatars (Cheskin and Kachuyevski, 2019). Thus, Russian language is especially prevalent (50% or more) in the eastern and regions of Ukraine such as Luhansk, Donetsk, Kharkiv, Kherson and Crimea, while the vast majority of the population in the west-Ukrainian regions such as Lviv, Vinnytsia and Khmelnytskyi prefer Ukrainian (State Statistics Committee of Ukraine, 2003; Kudriavtseva, 2021). In the central regions of Ukraine such as Cherkasy, many people speak so-called Surzhyk, a Russo-Ukrainian pidgin (Bilaniuk, 2004; Hentschel and Palinska, 2022). Furthermory, there are differences in the Ukrainian language, divided into northern, south-eastern and south-western dialects (Medynska et al., 2023). The first two groups can be located within Ukrainian-Belorussian and Ukrainian-Russian continua, the third group comprises of Volhynian–Podolian, Galician, Bukovynian and Transcarpathian dialects with Polish, Slovak, Hungarian and Romanian influences.

In summary, firstly, an east-west special gradient, and secondly, a temporal gradient from Russian to Ukrainian is apparent. Several Ukrainian dialects add to the complexity (Figueiredo and Silva, 2013; Figueiredo, 2019). Also the investigator needs to keep in mind that language always (Figueiredo and Silva, 2013; Figueiredo, 2019)—and even more so after 2022—has been a highly politicized matter in Ukraine, with Ukrainian being associated with expression of ethnic identity, national independence and a sense of cultural belonging (Palinska, 2023). Since Ukrainian currently undergoes rapid changes, with quickly developing military-related loanwords and neologisms (Del Gaudio, 2024), regular linguistic evaluations of any psychometric instrument will be required.

## 4 Theoretical framework and cross-cultural context: bipolar values

All these factors related to the previous linguistic background of refugees and asylum seekers must be carefully considered when evaluating the translation of a psychometric instrument. While it may not be feasible to develop a fully validated version for every dialect and cultural subgroup, it is crucial to acknowledge that individuals' understanding of specific items can vary depending on their cultural and linguistic backgrounds. Such variations may influence the outcomes and compromise the validity of the instrument. At a minimum, providing versions in both Ukrainian and Russian is essential for Ukrainian participants. A bilingual approach is critical to ensuring the psychometric validity of instruments, particularly when assessing trauma specificities and PTSD among Ukrainian refugees and asylum seekers.

Therefore, it is crucial to implement adjustments in the validation process with respect to word and semantic specifications, as well as syntax and phonology. Similar challenges arise with other instruments and languages, where variations in scoring across different populations introduce biases in reliability and inter-item total correlations (Poon et al., 2021). Specifically, for the group of Ukrainian refugees residing in or transitioning through various European countries, linguistic variations and pronounced semantic differentials are evident (Urbanaviče et al., 2024; Wylie et al., 2018). Regarding the semantic differential approach in psychology, it is essential to refer to the theoretical frameworks of “Control Affect Theory” or Osgood's “Atlas League” to better understand the semantic shifts in a cross-cultural context (Osgood, 1969; Robinson et al., 2006).

Affect is the central concept of this theory and varies according to word usage. According to Osgood's theory, the meaning associated with a word encompasses multiple affective traits that collectively contribute to its overall meaning (Hood et al., 2021; Osgood, 1969). These traits can be measured along three bipolar dimensions: evaluation, potency, and activity (Osgood, 1980, 2019). Affect encompasses emotional and sentimental features intrinsically linked to language. Consequently, Osgood's work is critical in this context, highlighting that conceptual understanding is not universal, which complicates word selection in psychometric tools. Furthermore, the translation and back-translation processes are influenced by the translators' and multidisciplinary research team's interpretations, shaped by their own languages and emotional frameworks (Dametto et al., 2023; Robinson et al., 2020).

Semantic differentials are founded on the principle that each meaning exists along a spectrum that must be examined and defined for a specific purpose. This purpose determines the appropriate understanding of what is intended to be conveyed within a particular community (Osgood, 2019). In the context of research and clinical assessment, constructs such as trauma and stress require precise definition across items and scales (Nielsen et al., 2023). We argue that identifying and measuring trauma necessitates evaluating dissociation in individuals, particularly regarding cognition, mood, and specific social interaction behaviors. However, this dissociation can be compromised during assessment if the instrument's items themselves are inconsistent or fragmented.

In psychometrics, semantics involves achieving linguistic consensus. Specifically, when addressing instruments like the PCL-5 and other trauma screening tools, the “bipolar value” of items (Osgood, 1969; Robinson et al., 2020) must be carefully considered. It is critical to ask: Do all the terms related to trauma within these scales maintain stability and consensus across the dimensions of “evaluation,” “potency,” and “activity,” as outlined in affect control theory? In Osgood's theory, evaluation refers to the degree to which an individual perceives a specific concept as positive or negative, as induced by words or keywords in speech. In the context of the PCL-5 items, we can estimate variations in the affective responses associated with the primary keywords that are tied to the experiences being assessed. These keywords often carry a strong negative affective value due to their connection with trauma-related stressors, such as those associated with war and pre-migration histories.

Potency pertains to the strength or impact of specific concepts as perceived by individuals, which is shaped by their cultural background and personal experiences. When responding to the PCL-5, participants may activate memories of recent experiences that resonate with high-potency words and expressions, particularly those related to themes of loss, grief, and traumatic events that currently hold significant relevance in their lives.

Activity, in Osgood's framework, relates to the frequency and intensity with which specific words or concepts are encountered and understood by individuals within their societal context. Responding to a scale like the PCL-5 involves interpreting items and their associated words or phrases, which vary in activity levels among participants depending on their exposure to traumatic events. These activity levels provide insights into the severity of stress responses and the progression toward PTSD.

Thus, the selection and presentation of words within the items of the PCL-5 are crucial. During the back-translation process, careful attention must be paid to ensure that the wording remains unbiased and that the original meaning of each item is preserved. Effective translation requires precise meaning transference, with curated choices of language and terminology to avoid any loss of the intended significance of the items. This ensures that the screening process remains reliable and culturally sensitive while accurately capturing the nuances of trauma-related experiences.

In this context, researchers, translators, and clinical practitioners involved in the adaptation or application of Ukrainian questionnaires to assess trauma should recognize the need for a standard that is currently unmet. Within the cross-cultural framework, the goal of translation is to preserve and convey the original meaning of each item cluster, ensuring that the scale remains reliable and replicable for repeated administration among speakers of a specific language (Poon et al., 2021; Ziegler and Bensch, 2013). However, cross-cultural comparisons are not feasible if the invariance of adapted instruments is not verified and reported by their authors.

In sum, by providing invariance data, including correlation coefficients, newly adapted versions of these instruments become valuable resources for both researchers and clinical practitioners (Forkus et al., 2023; Ziegler and Bensch, 2013). Moreover, comprehensive efforts in translation analysis are necessary to establish a standardized lexicon tailored to specific populations, particularly those with linguistic variations. This is especially relevant in the case of Ukrainian, where the language interacts with its regional dialects as well as Russian, necessitating careful consideration of linguistic nuances during translation and adaptation processes. Regarding setting, the research and clinical contexts use the same instruments for the trauma assessment but with some divergencies that we need to point out (Nielsen et al., 2023).

## 5 Semantic differential in trauma assessment: three PCL-5 versions in two languages across two populations of Ukrainian refugees

In line with the objective of the present study, we examined dissimilarities in differential semantic of two Ukrainian and one Russian versions of PCL-5 administered in two samples of Ukrainian refugees, in Germany and in Portugal, as compared to the original English version (Weathers et al., 2013a). Understanding the differences among the translated versions of the PCL-5 is crucial for evaluating how these variations may influence trauma assessments in Ukrainian refugees. To address this, we developed a combination of qualitative and quantitative methods as described below.

The qualitative method is grounded in content analysis, with a focus on conceptual equivalence and semantic differences within a cross-cultural framework. Specifically, this approach examines versions of the same instrument translated into the Ukrainian and Russian languages. For the language and psychological assessment verification, authors involved are fluent in languages of these versions. An inductive and reflexive thematic analysis was employed, in line with the guidelines proposed by Braun and Clarke (2021).

The quantitative method comprises a combination of expert rating of similarity between the back-translated PCL-5 versions to the English original with automated semantic similarity scores adapted from the machine translation field. To our knowledge, these techniques have never been employed in the fields of psychology and linguistic validation of translations of psychological instruments.

The German sample consists of data from 83 Ukrainian refugees (61 females, 22 males, age 39.5 ± 14.9 years (SD), 52.2% with had a higher education degree) who sought treatment in a large University outpatient clinic. Although the population comprised of residents from whole Ukraine, the majority of participants arrived from Kyiv, Kharkiv, Donetsk, Odessa, Dnipro, Kherson and Zaporizhzhia regions. All participants sought refuge in Germany following Russia's full-scale invasion 02/2022. This population had a high burden of psychiatric disease and symptoms, with 44.4% reporting a history of psychiatric illness, 42.8% fulfilling the DSM-5 A-criterion for trauma and a PTSD prevalence of 19.6%. They all underwent PCL-5 [52 in Russian (Russian version, RV, Los Angeles County, Department of Mental Health, 2019) and 31 in Ukrainian (UV1, Bezsheiko, 2016)] as part of their basic psychiatric assessment between 03/2022 and 12/2024. Before assessment, patients were asked for their language preference for this assessment.

In Portugal, 77 adult Ukrainian refugees (35 females, 42 males, age 44 ± 16.2 years, 79% with a higher education degree) participated in structured interviews using a Ukrainian version (Figueiredo et al., 2024) of the PTSD Checklist for DSM-5 (PCL-5; Weathers et al., 2013a). The individuals were originally from various Ukrainian cities, predominantly Mariupol, Kharkiv, and Kyiv. All participants arrived in Portugal with equal refugee status, after the onset of the Russian-Ukrainian conflict in February 2022 and were temporarily housed through a community reception program in the Lisbon metropolitan area. They are still residents in Portugal. Linguistically, all respondents were proficient in Ukrainian. Additionally, 48% reported fluency in Russian and 35% in English. Since the Ukrainian version of the PCL-5 used in the study had not previously undergone validation, no license was necessary. However, the instrument underwent review by a bilingual assistant fluent in both Ukrainian and Portuguese to ensure consistency with the original English version (Weathers et al., 2013b). The validation process was completed during earlier research (Figueiredo et al., 2024).

All responses were manually transcribed and coded by both researchers. This process was supported by prior guidance and input from the session team present during data collection. The coding and corresponding responses were subsequently reviewed collaboratively by the author team, and consensus was achieved. The qualitative data underwent reflexive thematic analysis following the six-phase process outlined by Braun and Clarke (2021), consisting of: (1) familiarization with the data, (2) generation of initial codes, (3) search for themes, (4) review of themes, (5) definition and naming of themes, and (6) final report production.

## 6 Qualitative semantic comparison of the three translations of PCL-5 to its English original

### 6.1 Methodology of the qualitative comparison

Initially, a back-translation of the two Ukrainian and the Russian versions of the PCL-5 into English was conducted using the DeepL translation program (DeepL SE, 2025). Subsequently, corrections were made by a native Ukrainian bilingual fluent in both Ukrainian and Russian, who ensured precise word-for-word accuracy. A bilingual psychologist proficient in Ukrainian and Russian refined these corrections. Please refer to in the Supplementary Table 1 for item-wise back-translation and the original version.

The analysis focused on word choices, semantic nuances, and inconsistencies identified across the translations as compared to the original English original. The emphasis was placed on preserving the original meaning inherent in the construct being assessed, ensuring that the translated items align with the conceptual framework of the PCL-5.

### 6.2 Examples of discrepancies

Supplementary Table 1 provides a detailed summary analysis of each PCL-5 item across the three translated versions, and with comparisons to the original version developed by Weathers et al. (2013a). At first glance, different terminology for the traumatic event becomes very expressive. In the original version (OV), as well as in the first Ukrainian version (UV1), it is referred to as “stressful experience”. In the second Ukrainian version (UV2) it is referred to it as a “stressful event/situation”, while in the Russian version (RV) it is named as “experienced stress”. These translations result in subtle semantic differences. A stressful experience, and even more so a stressful event/situation, implies one concrete, specific event, while “experienced stress” can be understood as a less acute and rather prolonged process, such as chronic life stress over several months and years. This can lead to profound misinterpretations of all items referring to this stress.

Differences in sentence structure also catch the eye. In the OV, all items are phrased as questions, omitting the verb like “are you” or “do you have”. The translated versions are less consistent in their structure. UV1 follows the original but items 16 and 17 step out of line. In the UV2 the items are not phrased as questions except 19 and 20. A part of the items is phrased addressing the 2nd person (“you”). In the RV, items are phrased as questions, but again, a part is phrased addressing the 2nd person. These structural inconsistencies within the scale can deteriorate its readability and understanding.

There are different translations for words like “upset” and “distressed”, or “anxiety”, “fear” and “horror” or “alertness” and “vigilance” or “detached”, “distant” and “separated” which are similar but have individual subtle semantic differences. In item 3 in the UV1 the explanatory sentence within the brackets is omitted, which may negatively impact the understanding of this item. In item 7 of the UV1, “conversations”, “actions” and “situations” are omitted in the description within the brackets. In item 9, the OV states “the world is completely dangerous”, which also occurs word by word in UV2. Item 9 in the RV includes “the world is very dangerous”, while the UV1 contains the statement “the world is a dangerous place”. These are semantically quite different statements and comprise gradations from “complete” to “very” to a simple “dangerous”, which will likely result in different scores for this item. In item 12, the RV translated “activities” as a “job” which obviously has a different meaning and may result in completely different scores. The translations of item 18 differ a lot between the versions. While it says “feeling jumpy or easily startled” in the OV, the translations range from “feelings of constant tension” to “anxiety or fearfulness” to “nervousness or anxiety”. These are different emotions, which are once again highly likely to result in different scores.

### 6.3 Summary of major discrepancies

Our comparison, with no precedents in this field, revealed that none of the 20 PCL-5 items across the three versions aligned entirely with the English original, compromising the integrity of the construct being assessed. Furthermore, the nuances of emotional expression were not consistently captured, showing lexical and semantic differences inherent to the translations. These discrepancies may undermine the reliability of emotional assessment, potentially affecting the individuals' ability to accurately convey their experiences (Shih, 2024; Zaid et al., 2021). Table 1 illustrates a summary of observed discrepancies in a color-dot scheme.

**Table 1:** Qualitative semantic discrepancy analysis of the three PCL-5 versions as compared to the English (expert rating).

**Item**	**UV1**	**UV2**	**RV**	**Discrepancy summary**
**1**	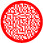	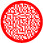	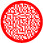	“Stress” used instead of “stressful experience”; but close to original
**2**	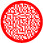	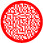	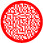	“Stress” used instead of “stressful experience”; but close to original
**3**	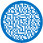	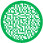	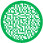	Loss of “reliving” nuance; phrasing issues, in UA1 > UA2 and RU
**4**	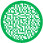	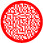	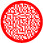	Tone/valence shift; vague “stress” in RU; UA2 and RU close to original
**5**	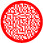	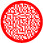	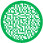	Minor variation in verbs; UA1 and UA2 closer to original than RU
**6**	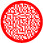	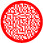	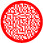	Minor stylistic differences; close to original
**7**	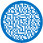	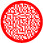	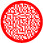	Significant variation in examples/list in UA1
**8**	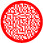	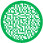	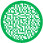	UA2 phrased as a statement; altered wording in RU
**9**	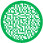	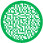	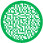	Slight wording alterations in all versions
**10**	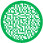	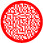	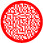	Slight wording alterations in UA1
**11**	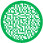	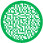	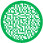	Minor variation in emotional terminology
**12**	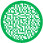	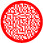	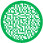	UA1/RU narrow to “job” or unspecified “activity(s)”; UA2 close to original
**13**	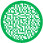	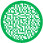	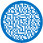	Tone variation: “remoteness” and “detachment” vs. “cut off”; in RU significant structural and semantic difference
**14**	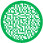	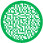	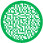	Slight differences in emotion expression
**15**	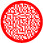	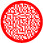	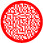	Fully aligned
**16**	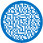	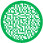	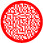	RU close to original, while minor to significantly altered description of risky behavior in UA2 and UA1
**17**	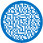	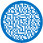	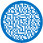	Different wording for “superalert” in each translation, major semantic differences to OV
**18**	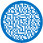	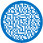	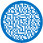	Different wording for “tension” in each translation, major semantic differences to OV
**19**	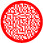	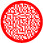	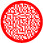	UA1 phrased as question; otherwise close matching
**20**	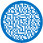	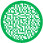	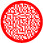	UA1 omits “staying asleep”; RU closest

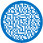
 = Score 1, significant semantic or structural discrepancy; 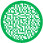 = Score 2, minor differences; 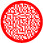 = Score 3, closely matches the English original.

The 20 items of the PCL-5 are designed to assess symptoms such as anxiety, intrusive memories, and abnormal daily behaviors. However, the observed linguistic and semantic variances particularly affect the wording associated with specific emotions, including the expression of emotional responses tied to the memory of traumatic experiences.

In sum, relatively high semantic incongruence regarding conceptual meaning was observed across all versions as compared to the original English original, posing a risk to the validity of the assessment and underscoring the necessity for meticulous translation and cultural adaptation processes.

## 7 Quantitative validation of the translations using machine metrics of semantic similarity and expert ratings

### 7.1 Automated similarity metrics for back-translation validation

We adapted four established text similarity metrics from machine translation evaluation to assess the degree of correspondence between original and back-translated psychometric items. Each metric addresses a different aspect of textual overlap:

- Levenshtein similarity—measures the minimum number of single-character edits (insertions, deletions, substitutions) required to transform one string into another (Levenshtein, 1966). We applied normalized similarity (1—distance/max length) after lowercasing, punctuation removal, spelling harmonization, and stemming (Porter, 1980). This is sensitive to literal changes and flags potentially meaningful wording differences in short, fixed-format items such as PCL-5.
- Damerau–Levenshtein similarity—extends Levenshtein by counting adjacent character transpositions as a single operation (Damerau, 1964), reducing penalty for common typographical errors. This provides a slightly more tolerant measure of literal similarity.
- Soft Cosine Similarity—unlike standard cosine similarity, which assumes words are orthogonal, Soft Cosine accounts for similarity between terms based on distributional semantics (Sidorov et al., 2014). We computed this using a Word2Vec model trained on the study corpus, enabling recognition of semantically related lexical choices (e.g., fear vs. terror) that may be equivalent in meaning but different in form.
- METEOR score—originally developed for MT evaluation (Banerjee and Lavie, 2005), METEOR combines exact and stem matches with precision–recall weighting and a fragmentation penalty to reward semantically correct but lexically varied translations. We adapted METEOR without synonym lists due to resource constraints, retaining exact/stem match, precision–recall weighting (α = 0.9), and fragmentation penalty (γ = 0.5).

Calculations were performed in customized Python scripts written with assistance of ChatGPT (OpenAI, 2025) using NumPy (Harris et al., 2020), Matplotlib (Hunter, 2007), Natural Language Toolkit (Loper and Bird, 2002), pandas (McKinney, 2010) and scikit-learn (Pedregosa et al., 2011) libraries.

For all metrics, scores range from zero (no similarity) to one (identical). In our context, ≥0.95 indicates near-identical form, 0.85–0.94 high similarity with minor wording differences, 0.70–0.84 moderate similarity potentially affecting nuance, and < 0.70 low similarity warranting careful review for conceptual equivalence.

Statistical analysis was performed in Python. Similarity metrics and expert scores across the three back-translated versions were analyzed using one-factor ANOVA. Spearman's rank correlation was used for analysis of correlation between similarity metrics and expert scores. All values are presented as mean ± standard error of the mean if not stated otherwise.

### 7.2 Expert evaluation

Two experts scored similarity of each back-translated item to its original on a scale from one to three; with a score of one being the low similarity indicating significant semantic or structural discrepancy; two indicating medium similarity with some differences but closely matching the English original; and a score of three indicating high similarity with minor or no differences.

### 7.3 Results of the quantitative validation

Please refer to Table 2 for item-wise expert ratings and automated similarity metrics. In the expert rating, translated versions scored between 2.05 ± 0.18 (UV1) to 2.35 ± 0.17 (RV) and 2.40 ± 0.15 (UV2), indicating mediocre similarity with possible semantic or structural discrepancies to the original. Albeit ANOVA revealed no statistical difference in the expert ratings between these translations [*F*_(2, 57)_ = 1.27, *p* = 0.29], low scores in domain E of PCL-5 strike the eye especially in UV1 (1.67) and UV2 (2.00), while the highest scores prevail in domain B, ranging from 2.40 in UV1 to 2.80 to UV2 and in domain C (a score of 3.00 in both, OV2 and RV).

**Table 2:** Quantitative semantic discrepancy analysis of the three PCL-5 versions as compared to the English original using four linguistic similarity metrics and expert ratings.

**Domain**	**Item**	**Damerau–Levenshtein**			**Levenshtein**			**METEOR**			**Soft Cosine**			**Expert rating**		
		**UV1**	**UV2**	**RV**	**UV1**	**UV2**	**RV**	**UV1**	**UV2**	**RV**	**UV1**	**UV2**	**RV**	**UV1**	**UV2**	**RV**
**B**	1	0.77	0.77	0.89	0.71	0.76	0.85	0.74	0.62	0.75	0.82	0.90	0.85	3	3	3
	2	0.75	0.71	0.76	0.78	0.70	0.75	0.49	0.49	0.67	0.61	0.84	0.56	3	3	3
	3	0.51	0.49	0.44	0.49	0.51	0.47	0.73	0.78	0.70	0.83	0.81	0.78	1	2	2
	4	0.72	0.41	0.92	0.66	0.42	0.91	0.89	0.59	0.46	0.75	0.76	0.97	2	3	3
	5	0.78	0.61	0.77	0.73	0.56	0.72	0.69	0.75	0.54	0.87	0.82	0.88	3	3	2
**Mean (B)**		**0.71**	**0.60**	**0.75**	**0.67**	**0.59**	**0.74**	**0.71**	**0.65**	**0.62**	**0.78**	**0.82**	**0.81**	**2.40**	**2.80**	**2.60**
**C**	6	0.69	0.81	0.80	0.67	0.80	0.81	0.64	0.55	0.82	0.83	0.95	0.88	3	3	3
	7	0.28	0.34	0.28	0.27	0.36	0.31	0.60	0.46	0.56	0.76	0.85	0.83	1	3	3
**Mean (C)**		**0.48**	**0.58**	**0.54**	**0.47**	**0.58**	**0.56**	**0.62**	**0.51**	**0.69**	**0.80**	**0.90**	**0.85**	**2.00**	**3.00**	**3.00**
**D**	8	0.71	0.44	0.90	0.64	0.47	0.92	0.75	0.68	0.31	0.71	0.57	0.91	3	2	2
	9	0.80	0.64	0.75	0.73	0.62	0.69	0.76	0.85	0.63	0.94	0.88	0.89	2	2	2
	10	0.61	0.70	0.66	0.56	0.70	0.65	0.62	0.66	0.78	0.86	0.91	0.88	2	3	3
	11	0.74	0.63	0.67	0.71	0.63	0.66	0.68	0.68	0.67	0.85	0.78	0.81	2	2	2
	12	0.64	0.56	0.64	0.61	0.52	0.63	0.64	0.56	0.46	0.67	0.52	0.75	2	3	2
	13	0.80	0.59	0.65	0.84	0.51	0.62	0.44	0.75	0.38	0.92	0.70	0.69	2	2	1
	14	0.63	0.61	0.51	0.64	0.63	0.45	0.46	0.54	0.61	0.79	0.76	0.60	2	2	2
**Mean(D)**		**0.70**	**0.60**	**0.68**	**0.68**	**0.58**	**0.66**	**0.62**	**0.67**	**0.55**	**0.82**	**0.73**	**0.79**	**2.14**	**2.29**	**2.00**
**E**	15	0.93	0.77	0.36	0.93	0.78	0.45	0.25	0.82	0.97	0.95	0.93	0.85	3	3	3
	16	0.39	0.56	0.56	0.35	0.57	0.57	0.47	0.18	0.47	0.49	0.73	0.74	1	2	3
	17	0.41	0.27	0.36	0.33	0.26	0.33	0.28	0.65	0.11	0.74	0.46	0.62	1	1	1
	18	0.39	0.46	0.19	0.36	0.53	0.30	0.10	0.30	0.49	0.46	0.61	0.35	1	1	1
	19	0.45	0.63	0.58	0.51	0.69	0.47	0.50	0.47	0.50	0.60	0.64	0.66	3	3	3
	20	0.33	0.64	0.36	0.30	0.72	0.48	0.26	0.33	0.51	0.46	0.68	0.38	1	2	3
**Mean(E)**		**0.48**	**0.55**	**0.40**	**0.46**	**0.59**	**0.43**	**0.31**	**0.46**	**0.51**	**0.62**	**0.67**	**0.60**	**1.67**	**2.00**	**2.33**
**Mean±SD**		**0.62**	**0.58**	**0.60**	**0.59**	**0.59**	**0.60**	**0.55**	**0.58**	**0.57**	**0.75**	**0.75**	**0.74**	**2.05**	**2.40**	**2.35**
**(overall)**		**±0.19**	**±0.14**	**±0.22**	**±0.19**	**±0.14**	**±0.19**	**±0.21**	**±0.18**	**±0.19**	**±0.15**	**±0.14**	**±0.17**	**±0.83**	**±0.68**	**±0.75**

Legend: UV1, Ukrainian version 1; UV2, Ukrainian version 2; RV, Russian version. Expert rating: 1 (red), significant semantic or structural discrepancy; 2 (blue), matches the English original with possible semantic or structural discrepancy; 3 (black), minor or no differences. The linguistic metric scores range from 0 (no overlap) to 1 (maximum overlap). For all metrics, scores above 0.7 are considered acceptable, indicating at least moderate similarity with likely preserved meaning. Bold values represent mean values or standard deviations.

Damerau–Levenshtein, Levenshtein and METEOR scores all ranged between 0.55 and 0.62, while Soft Cosine scores ranged between 0.74 and 0.75. These metrics indicate low to moderate similarity to the original, with a relatively high likelihood for loss of nuance and semantic differences. These scores did not differ significantly amongst translations [*F*_(2, 57)_ = 0.18, *p* = 0.84; *F*_(2, 57)_ = 0.04, *p* = 0.96; *F*_(2, 57)_ = 0.18, *p* = 0.84; *F*_(2, 57)_ = 0.03, *p* = 0.97, respectively]. Similarly to expert ratings, lowest scores were found in domain E (0.31–0.59 in the first three methods and 0.60–0.67 in Soft Cosine]. Apparently, translation of items regarding trauma-related arousal and hyperreactivity poses a linguistic challenge.

Then we calculated Spearman's rank correlation (ρ) between expert ratings and semantic discrepancy scores (Table 3). For all three back-translations, we found a strong correlation between expert ratings and Damerau–Levenshtein and Levenshtein scores (ρ between 0.59 and 0.75, *p* < 0.01 to *p* < 0.001). For METEOR and Soft Cosine the correlation with expert ratings was weak and statistically insignificant (ρ between 0.17 and 0.34, *p* > 0.1). Thus, Damerau–Levenshtein and Levenshtein, but not METEOR nor Soft Cosine, seem suitable for semantic validation of translations of psychological instruments.

**Table 3:** Spearman's rank correlation (ρ) between expert rating and semantic discrepancy scores with corresponding *p*-values for each of the three PCL-5 versions.

**Method**	**UV1**		**UV2**		**RV**	
	**ρ**	***p***	**ρ**	***p***	**ρ**	***p***
Damerau–Levenshtein	0.69	8 × 10^−4^	0.59	5.7 × 10^−3^	0.75	1.2 × 10^−4^
Levenshtein	0.73	2.9 × 10^−4^	0.62	3.9 × 10^−3^	0.75	1.2 × 10^−4^
METEOR	0.32	0.17	0.26	0.26	0.23	0.31
Soft Cosine	0.32	0.17	0.23	0.33	0.22	0.34

## 8 Conclusion and practical implications

Our qualitative and quantitative analyses both revealed merely mediocre semantic and structural overlap across the four versions of the PCL-5 examined. We found no significant differences in the translation quality overall, albeit a slight tendency in preference toward UV2 over UV1 can be stated. Some of the automated linguistic similarity metrics employed, particularly Levenshtein and Damerau–Levenshtein, highly correlate with expert ratings, possibly presenting a novel tool for the validation process.

The cultural significance of specific words varies considerably, posing challenges to the assessment process, particularly when relying on the written templates used. Errors can occur during the translation process, both at the lexical and syntactic levels, especially during back-translation. Cultural factors are pivotal in evaluating constructs that are not directly observable. Refugees within a specific societal and linguistic context may encounter difficulties in comprehending items on psychological instruments like the PCL-5. Furthermore, the construct measured by the instrument may undergo alterations that significantly affect the intended measurement objectives.

Due to the lack of validation studies on the investigated Ukrainian and Russian translations of the PCL-5, linguistic and structural inconsistencies and cultural adjustments are difficult to identify. Three key issues—cultural meaning mismatches, biased construct measurement (Figueiredo, 2024), and a lack of data on and monitoring of the translation process—undermine the psychometric properties and the accuracy of the PCL-5 for specific displaced groups such as Ukrainians.

Thus, our future research will be focused on the development of validated Ukrainian and Russian translations of the PCL-5.

Despite these concerns, the PCL-5 is a powerful and efficient instrument for assessment of PTSD symptoms, and we advise the (cautious) use of translations available amongst Ukrainian refugees until potentially improved, validated versions are developed, following clinical guidelines below:

1. Prior to administering PCL-5, assess whether the DSM trauma-criterion for PTSD is fulfilled (e.g., using the LEC) to decrease false-positive rates.
2. Pay attention to cultural and linguistic sensitivity (sociodemographic background of your sample, their language preferences, time of arrival in the host country, psychological burden).
3. Offer PCL-5 and all other instruments in the language of their choice.
4. Clarify patient's questions und uncertainties regarding the meaning of any item.
5. Consider different cut-offs for different populations and purposes (lower cut-off around 30 makes sense for screening purposes (high sensitivity, low specificity) in a general population; higher cut-off (34 and above) can be implemented to increase specificity in a sample with high psychological burden).
6. Complement PCL-5 with instruments measuring symptoms of depression and anxiety such as BDI-II, MADRS and BAI.
7. Never base your clinical diagnosis on an instrument alone, but on a synthesis of the patient's history, your psychological assessment (in presence of a translator fluent in both languages) and test results.
8. Consider differential diagnoses of PTSD such as major depression and personality disorders.

## 9 Limitations

We acknowledged limitations of the PCL-5 itself in paragraph 2.2 and limitations regarding the absence of psychometric validation of the translated versions in paragraph 3.2 of this data report. A major limitation lies in the use of non-standardized translations sourced from public repositories, which were the only versions available. Nonetheless, psychometric properties of UV2 have already been studied by the first author (Figueiredo et al., 2024). The automated back-translation tool employed (DeepL) may not meet the rigorous standards required for formal linguistic validation. Expert ratings are intrinsically vulnerable to subjective variance. The use of automated semantic similarity measures is a novel experimental approach and needs further refinement and validation.

## Data Availability

The raw data supporting the conclusions of this article will be made available by the authors, without undue reservation.
