# Supplementary material for: PTSD assessment in Ukrainian refugees using the PCL-5: linguistic and cultural challenges
Source: Front Psychol. 2025 Sep 16;16:1659301. doi: 10.3389/fpsyg.2025.1659301 (PMC12479293; doi:10.3389/fpsyg.2025.1659301)
Supplement: Supplementary file 1 [file Table_1.docx]

**Supplemental Table 1**

Original PCL-5 in English and back-translations of the two Ukrainian and one Russian version

| Item | Ukrainian 1  (Bezsheiko, 2016) | Ukrainian 2  (Oxford Health NHS Foundation; Figueiredo et al., 2024) | Russian  (Los Angeles, Dep. of Mental Health) | English original  (Weathers et al., 2013a) |
| --- | --- | --- | --- | --- |
| 1 | Repetitive, disturbing and unwanted memories of stressful experiences? | Repeated, disturbing and unwanted memories of the stressful event/situation | Repetitive, disturbing and unwanted memories of experienced stress? | Repeated, disturbing, and unwanted memories of the stressful experience? |
| 2 | Recurring, disturbing dreams about stressful experiences? | Recurrent, disturbing dreams about the stressful experience/situation | Recurring, disturbing dreams about experienced stress? | Repeated, disturbing dreams of the stressful experience? |
| 3 | Sudden feelings or behaviour as if the stressful experience is happening again? | Suddenly feeling or behaving as if the stressful event/situation is happening again (as if you are actually experiencing it again) | Sudden feeling or behaving as if the stress you experienced happened again (as if you actually went back and experienced it again)? | Suddenly feeling or acting as if the stressful experience were actually happening again (as if you were actually back there reliving it)? |
| 4 | Feeling upset when something reminds you of the stressful experience? | Being very upset if something reminds you of the stressful event/situation | Feeling very distressed when reminded of the experienced stress? | Feeling very upset when something reminded you of the stressful experience? |
| 5 | Strong physical reactions when something reminded you of the stressful experience (e.g. heart palpitations, shortness of breath, sweating)? | Feeling strong physical reactions when something reminds you of the stressful event/situation (e.g., heart palpitations, breathing problems, sweating) | Strong physical reactions when reminded of the experienced stress? (e.g. rapid heartbeat, difficulty breathing, sweating)? | Having strong physical reactions when something reminded you of the stressful experience (for example, heart pounding, trouble breathing, sweating)? |
| 6 | Avoidance of memories, thoughts or feelings associated with the stressful experience? | Avoiding memories, thoughts or feelings related to the stressful event/situation | Avoidance of memories, thoughts or feelings associated with the experienced stress? | Avoiding memories, thoughts, or feelings related to the stressful experience? |
| 7 | Avoidance of external stimuli (people, objects, places...) that remind you of the stressful experience? | Avoiding external reminders of stressful events/situations (e.g., people, places, conversations, actions, objects, or situations) | Avoidance of external reminders of the experienced stress (e.g., people, places, dialogues, actions, objects or situations)? | Avoiding external reminders of the stressful experience (for example, people, places, conversations, activities, objects, or situations)? |
| 8 | Trouble remembering important moments of the stressful experience? | You cannot remember important moments of the stressful event/situation | Difficulties in recalling important components of the experienced stress? | Trouble remembering important parts of the stressful experience? |
| 9 | Strong negative beliefs about yourself, other people, or the world around you (e.g., ‘I am bad’, ‘there is something very wrong with me’, ‘no one can be trusted’, ‘the world is a dangerous place’)? | You feel strong negative beliefs about yourself, other people, or the world (e.g., thoughts such as: I am bad, there is something very wrong with me, no one can be trusted, the world is completely dangerous) | Strong negative beliefs about yourself, others, or the world (e.g., having thoughts such as: I'm bad, there's something wrong with me, I can't trust anyone, the world is very dangerous)? | Having strong negative beliefs about yourself, other people, or the world (for example, having thoughts such as: I am bad, there is something seriously wrong with me, no one can be trusted, the world is completely dangerous)? |
| 10 | Self-blame or blaming others for the stressful experience or what happened afterwards? | Blaming yourself or someone else for the stressful event/situation or for what happened afterwards | Blaming yourself or others for the stress you experienced or for what happened afterwards? | Blaming yourself or someone else for the stressful experience or what happened after it? |
| 11 | Strong negative emotions such as anxiety, fear, anger, guilt or shame? | Strong negative feelings such as fear, disgust, anger, guilt or shame | Presence of strong negative feelings, such as fear, terror, anger, guilt or shame? | Having strong negative feelings such as fear, horror, anger, guilt, or shame? |
| 12 | Loss of interest in the activity(s) that used to bring pleasure? | Loss of interest in activities you used to enjoy | Loss of interest in a job you used to enjoy? | Loss of interest in activities that you used to enjoy? |
| 13 | Feelings of remoteness or separation from other people? | Feelings of remoteness or disconnection from other people | A sense of detachment from other people? | Feeling distant or cut off from other people? |
| 14 | Problems in experiencing positive emotions (e.g., inability to feel joy or love towards a close person) | Difficulties with experiencing positive feelings (e.g., inability to feel happiness or love for close people) | Difficulties experiencing positive feelings (e.g. inability to feel joy or love towards close people)? | Trouble experiencing positive feelings (for example, being unable to feel happiness or have loving feelings for people close to you)? |
| 15 | Irritability, outbursts of anger, aggressive behaviour? | Irritability, outbursts of anger or aggressive behaviour | Irritable behaviour, outbursts of anger or aggressive behaviour? | Irritable behaviour, angry outbursts, or acting aggressively? |
| 16 | That you're taking a lot of risks or doing things that could be harmful? | Too much risky behaviour or actions that could cause you harm | Taking too many risks or doing things that could bring you harm? | Taking too many risks or doing things that could cause you harm? |
| 17 | To be ‘on the alert’ or ‘on the lookout’? | A state of ‘high alert’ or vigilance or being on the alert | A state of ‘super vigilance’ or wariness or heightened vigilance? | Being “superalert” or watchful or on guard? |
| 18 | A feeling of constant tension? | Feeling of anxiety or fearfulness | A feeling of nervousness or anxiety? | Feeling jumpy or easily startled? |
| 19 | Difficulties with concentration? | Do you have difficulties concentrating? | Problems with concentration? | Having difficulty concentrating? |
| 20 | Problems falling asleep or waking up at night? | Difficulty sleeping or falling asleep? | Problems falling asleep or staying asleep? | Trouble falling or staying asleep? |

Legend: OV, original version; UV1, Ukranian version 1; UV2, Ukranian version 2; RV, Russian version.
